# Editorial: Methods in cancer endocrinology

**DOI:** 10.3389/fendo.2022.1096028

**Published:** 2022-12-05

**Authors:** Chiara Martinelli, Carlotta Pucci

**Affiliations:** ^1^ Department of Chemistry, Materials and Chemical Engineering “Giulio Natta”, Politecnico di Milano, Milan, Italy; ^2^ Smart Bio-Interfaces, Istituto Italiano di Tecnologia, Pontedera, Italy

**Keywords:** diagnosis, prognosis, methods, biomarkers, thyroid cancer

Cancer Endocrinology is a broad discipline focused on the study of endocrine tumors, from the ones directly related to endocrine glands and neuroendocrine system to cancers involving other organs and tissues indirectly connected to the endocrine system. Finding new strategies for diagnosis, studying specific molecular pathways, and developing new therapies for giving a better clinical prognosis to affected patients are only a few of the most important objectives in this field of research.

The present Research Topic aims to provide a collection of experimental techniques and methods for diagnosis and prognosis in this field. More specifically, the Research Topic includes four original research papers providing insights into new methods, biomarkers and models that can be used in the prediction, diagnosis, and treatment of thyroid cancers.

Differentiated thyroid carcinoma (DTC) is the most common thyroid malignant tumor subjected to established standard therapies (1). Between the biomarkers selected for patient’s follow-up there is thyroglobulin, that supports disease surveillance (2). The stimulated thyroglobulin is currently considered as an efficient predictive value; however, measurements can be affected by many factors, thus reducing its validity (3, Wang et al. ). The antithyroglobulin antibody, an autoantibody against thyroglobulin, is commonly detected in DTC patients (4), yet it can interfere with a correct thyroglobulin evaluation (5, 6). The first article in the present Research Topic by Pan et al. aimed at analyzing the combination between stimulated thyroglobulin and antithyroglobulin antibody in predicting the efficacy and prognosis of therapy with radioactive iodine in patients affected by differentiated thyroid carcinomas after total thyroidectomy and independently from their positivity or negativity for antithyroglobulin antibody. This strategy revealed successful in patients subjected to surgery and before the initial radioactive iodine treatment, demonstrating to be a valid surveillance indicator in the clinical context.

N6 methyladenosine (m6A) abnormal modifications have been linked to several dysfunctions, such as tumorigenesis, neurological system diseases, embryonic developmental disorders, immune cell homeostasis and differentiation failure (7–9). The implications of m6A modifications in tumor progression have been highlighted for different kinds of tumors; however, its role in papillary thyroid carcinoma (PTC) needs to be better investigated. In particular, its correlation with the fat mass and obesity-associated proteins (FTO) has not been clarified. FTO is known to catalyze the demethylation of m6A (10) and its knockdown can enhance m6A upregulation. In the second research paper in this Research Topic, Ji et al. demonstrated how FTO can inhibit the formation of PTC by downregulating the expression of SLC7A11 through ferroptosis. The results shown in this work might help in finding new biomarkers and/or therapeutic targets for PTC and they could also give new insights in the elucidation of the relationship between tumor malignancy and m6A.

Medullary thyroid cancer (MTC) is a malignant tumor accounting for 1%-2% of all thyroid cancers (11). This tumor is characterized by calcitonin secretion (12), a recognized prognostic marker evaluated after initial surgical treatment, a procedure whose extent remains yet divisive (13, 14). This is mainly related to the decision on whether patients without evident lateral lymph node metastases are recommended or not undergoing lateral neck dissection (15). The third article in the present Research Topic by Jin et al. studied a multivariate logistic regression model based on several prognostic factors. A nomogram of the proposed model was proposed for predicting lateral lymph node metastases in medullary thyroid cancer patients. The research demonstrated the involvement of some specific risk factors in this pathology and evidenced the accuracy of the prediction in selecting patients for which lateral neck dissection is recommended.

PTC comprises 80% – 85% of all thyroid cancers (16); among these, around 40% – 50% of PTC is composed of papillary thyroid microcarcinomas (PTMC) (17). PTMC treatment strategy depends on the aggressiveness of the specific patient’s tumor; for example, high-risk PTMC, characterized by an extremely aggressive tumor phenotype, with local recurrences and metastasis, is treated with strong therapeutic approaches, including surgical resection, when possible. However, some low-risk PTMC also show recurrences, resulting in poor prognosis if not treated properly. Therefore, a better prediction marker for tumor aggressiveness in PTMC must be found to better select the correct therapeutic strategy. Mutations at the level of the BRAF gene have been linked to PTMC recurrence and metastasis and can be, thus, used for monitoring low-risk PTMC (18). Fine-needle aspiration (FNA) is commonly used to diagnose BRAF mutation in preoperative identification (19); however, FNA is not recommended for nodules with a diameter lower than 1 cm (20). The fourth article in the present Research Topic by Tang et al. proposes a new method for the prediction of preoperative BRAF mutation for PTMC patients, by means of a specific ultrasound (US) radiomics nomogram developed by the authors. The US radiomics nomogram showed higher discriminative ability than the conventional US model and can be a precious tool for the selection of the best therapeutic strategy for PTMC patients.

In conclusion, this Research Topic presents four different original research articles with the common aim of highlighting new avenues in the diagnosis and treatment of thyroid cancers, paving the way for the introduction of novel and more efficient methods in the clinical practice.

## Author contributions

Conceptualization and Writing: CM and CP; Review and Editing: CM and CP. All authors contributed to the article and approved the submitted version.

## References

[1] Mazzaferri EL Kloos RT . Clinical review 128: Current approaches to primary therapy for papillary and follicular thyroid cancer. J Clin Endocrinol Metab (2001) 86:1447–63. doi: 10.1210/jcem.86.4.7407 11297567

[2] Kendler DB Vaisman F Corbo R Martins R Vaisman M . Preablation stimulated thyroglobulin is a good predictor of successful ablation in patients with differentiated thyroid cancer. Clin Nucl Med (2012) 37:545–9. doi: 10.1097/RLU.0b013e31824852f8 22614184

[3] Wang C Diao H Ren P Wang X Wang Y Zhao W . Efficacy and affecting factors of (131)I thyroid remnant ablation after surgical treatment of differentiated thyroid carcinoma. Front Oncol (2018) 8:640. doi: 10.3389/fonc.2018.00640 30619772 PMC6306449

[4] Spencer C Fatemi S . Thyroglobulin antibody (TgAb) methods - strengths, pitfalls and clinical utility for monitoring TgAb-positive patients with differentiated thyroid cancer. Best Pract Res Clin Endocrinol Metab (2013) 27:701–12. doi: 10.1016/j.beem.2013.07.003 24094640

[5] Spencer CA Takeuchi M Kazarosyan M Wang CC Guttler RB Singer PA . Serum thyroglobulin autoantibodies: prevalence, influence on serum thyroglobulin measurement, and prognostic significance in patients with differentiated thyroid carcinoma. J Clin Endocrinol Metab (1998) 83:1121–7. doi: 10.1210/jcem.83.4.4683 9543128

[6] Spencer C Petrovic I Fatemi S . Current thyroglobulin autoantibody (TgAb) assays often fail to detect interfering TgAb that can result in the reporting of falsely low/undetectable serum tg IMA values for patients with differentiated thyroid cancer. J Clin Endocrinol Metab (2011) 96:1283–91. doi: 10.1210/jc.2010-2762 21325460

[7] Ma JZ Yang F Zhou CC Liu F Yuan JH Wang F . METTL14 suppresses the metastatic potential of hepatocellular carcinoma by modulating N6-methyladenosine-dependent primary MicroRNA processing. Hepatology (2017) 65:529–43. doi: 10.1002/HEP.28885/SUPPINFO 27774652

[8] Chen M Wei L Law CT Tsang FHC Shen J Cheng CLH . RNA N6-methyladenosine methyltransferase-like 3 promotes liver cancer progression through YTHDF2-dependent posttranscriptional silencing of SOCS2. Hepatology (2018) 67:2254–70. doi: 10.1002/HEP.29683/SUPPINFO 29171881

[9] Chen Y Peng C Chen J Chen D Yang B He B . WTAP facilitates progression of hepatocellular carcinoma *via* m6A-HuR-dependent epigenetic silencing of ETS1. Mol Cancer (2019) 18:1–19. doi: 10.1186/S12943-019-1053-8/FIGURES/7 31438961 PMC6704583

[10] Jia G Fu Y Zhao X Dai Q Zheng G Yang Y . N6-methyladenosine in nuclear RNA is a major substrate of the obesity-associated FTO. Nat Chem Biol (2011) 7:885–7. doi: 10.1038/nchembio.687 PMC3218240 22002720

[11] Cooper MR Yi SY Alghamdi W Shaheen DJ Steinberg M . Vandetanib for the treatment of medullary thyroid carcinoma. Ann Pharmacother (2014) 48:387–94. doi: 10.1177/1060028013512791 24259657

[12] Roman S Lin R Sosa JA . Prognosis of medullary thyroid carcinoma: demographic, clinical, and pathologic predictors of survival in 1252 cases. Cancer (2006) 107:2134–42. doi: 10.1002/cncr.22244 17019736

[13] Wells SAJ Asa SL Dralle H Elisei R Evans DB Gagel RF . Revised American thyroid association guidelines for the management of medullary thyroid carcinoma. Thyroid (2015) 25:567–610. doi: 10.1089/thy.2014.0335 25810047 PMC4490627

[14] Siironen P Hagström J Mäenpää HO Louhimo J Arola J Haglund C . Lymph node metastases and elevated postoperative calcitonin: Predictors of poor survival in medullary thyroid carcinoma. Acta Oncol (2016) 55:357–64. doi: 10.3109/0284186X.2015.1070963 26339947

[15] Asarkar A Chang BA Nathan C-AO . What is the extent of neck dissection in medullary thyroid carcinoma? Laryngoscope (2021) 131:458–9. doi: 10.1002/lary.28686 32311764

[16] Livolsi VA . Papillary thyroid carcinoma: an update. Mod Pathol 2011 242 (2011) 24:S1–9. doi: 10.1038/modpathol.2010.129 21455196

[17] Seib CD Sosa JA . Evolving understanding of the epidemiology of thyroid cancer. Endocrinol Metab Clin North Am (2019) 48:23–35. doi: 10.1016/J.ECL.2018.10.002 30717905

[18] Kim KJ Kim SG Tan J Shen X Viola D Elisei R . BRAF V600E status may facilitate decision-making on active surveillance of low-risk papillary thyroid microcarcinoma. Eur J Cancer (2020) 124:161–9. doi: 10.1016/J.EJCA.2019.10.017 31790974

[19] Di Benedetto G Fabozzi A Rinaldi C Rinaldi CR . BRAF test and cytological diagnosis with a single fine needle cytology sample. Acta Cytol (2013) 57:337–40. doi: 10.1159/000350618 23860494

[20] Tessler FN Middleton WD Grant EG Hoang JK Berland LL Teefey SA . ACR thyroid imaging, reporting and data system (TI-RADS): White paper of the ACR TI-RADS committee. J Am Coll Radiol (2017) 14:587–95. doi: 10.1016/j.jacr.2017.01.046 28372962

